# In Situ 3D Printing of Conformal Bioflexible Electronics via Annealing PEDOT:PSS/PVA Composite Bio-Ink

**DOI:** 10.3390/polym17111479

**Published:** 2025-05-26

**Authors:** Xuegui Zhang, Chengbang Lu, Yunxiang Zhang, Zixi Cai, Yingning He, Xiangyu Liang

**Affiliations:** 1 School of Physics and Optoelectronics, Xiangtan University, Xiangtan 411105, China; 202221521380@smail.xtu.edu.cn (X.Z.); 202321521387@smail.xtu.edu.cn (Y.Z.); 2 Agricultural Genomics Institute at Shenzhen, Chinese Academy of Agricultural Sciences, Shenzhen 518120, China; luchengbang@caas.cn (C.L.); caizixi@mail.nwpu.edu.cn (Z.C.); 3 Institute of Bast Fiber Crops and Center of Southern Economic Crops, Chinese Academy of Agricultural Sciences, Changsha 410205, China; 4 State Key Laboratory of Molecular Engineering of Polymers, Department of Macromolecular Science, Fudan University, Shanghai 200438, China

**Keywords:** 3D printing, bioelectronics, bio-ink, flexible sensors, direct ink writing

## Abstract

High-performance flexible sensors capable of direct integration with biological tissues are essential for personalized health monitoring, assistive rehabilitation, and human–machine interaction. However, conventional devices face significant challenges in achieving conformal integration with biological surfaces, along with sufficient biomechanical compatibility and biocompatibility. This research presents an in situ 3D biomanufacturing strategy utilizing Direct Ink Writing (DIW) technology to fabricate functional bioelectronic interfaces directly onto human skin, based on a novel annealing PEDOT:PSS/PVA composite bio-ink. Central to this strategy is the utilization of a novel annealing PEDOT:PSS/PVA composite material, subjected to specialized processing involving freeze-drying and subsequent thermal annealing, which is then formulated into a DIW ink exhibiting excellent printability. Owing to the enhanced network structure resulting from this unique fabrication process, films derived from this composite material exhibit favorable electrical conductivity (ca. 6 S/m in the dry state and 2 S/m when swollen) and excellent mechanical stretchability (maximum strain reaching 170%). The material also demonstrates good adhesion to biological interfaces and high-fidelity printability. Devices fabricated using this material achieved good conformal integration onto a finger joint and demonstrated strain-sensitive, repeatable responses during joint flexion and extension, capable of effectively transducing local strain into real-time electrical resistance signals. This study validates the feasibility of using the DIW biomanufacturing technique with this novel material for the direct on-body fabrication of functional sensors. It offers new material and manufacturing paradigms for developing highly customized and seamlessly integrated bioelectronic devices.

## 1. Introduction

Leveraging their portability and real-time monitoring capabilities, wearable electronics are profoundly transforming multiple frontier fields, including health management and virtual reality. In health management, these devices provide continuous streams of physiological data, opening new avenues for personalized medicine, early disease warning, and chronic disease management; conversely, in virtual reality and augmented reality applications, they significantly enhance the immersion and naturalness of human–machine interaction by capturing users’ subtle actions and physiological feedback. Across all these application scenarios, sensors capable of real-time, accurate perception of human physiological signals and dynamic states are undoubtedly essential functional components [1,2,3,4,5,6]. Establishing stable, conformal, and functional bioelectronic interfaces between these devices and human tissues is critical for their efficacy [7,8,9,10]. Such interfaces must not only ensure reliable signal acquisition and transmission but also seamlessly conform to complex body surfaces, sustain their sensing function across diverse physiological environments, and critically address biosafety considerations. Flexible sensors, particularly those capable of precisely capturing subtle human movements or physiological deformations, offer broad utility in rehabilitation engineering [11], sports science [12], and assistive technologies requiring precise human–computer interaction. Consequently, these sensors must possess good biomechanical compatibility to accommodate dynamic tissue changes [13,14,15].

However, conventional manufacturing approaches for electronic devices typically struggle to meet the requirements for fabricating high-performance sensors directly onto soft, irregular, and dynamically changing biological surfaces. Rigid devices fail to achieve conformal contact. While pre-fabricated flexible devices represent an improvement, their interfacial integration with biological tissues often relies on adhesives that can cause irritation or discomfort. Furthermore, the complex fabrication processes involved restrict personalization and on-demand manufacturing [16].

Furthermore, functional materials utilized for constructing high-performance flexible bioelectronic devices often exhibit inherent performance limitations, primarily because ideal devices require not only efficient signal transduction and transmission capabilities but also excellent mechanical flexibility to accommodate the dynamic nature of biological tissues, coupled with good biocompatibility for safe application; however, single materials typically struggle to perfectly reconcile these often conflicting requirements. The conductive polymer Poly(3,4-ethylenedioxythiophene): poly(styrenesulfonic acid) (PEDOT:PSS), for instance, is extensively studied owing to its excellent room-temperature conductivity and good solution processability. However, its films typically exhibit significant brittleness, with an elongation at break often below 5% and a relatively high Young’s modulus, meaning it is prone to fracture or micro-cracking even under small deformations. Polyvinyl alcohol (PVA), on the other hand, can provide ideal mechanical support that better matches soft biological tissues [17,18,19], tunable mechanical strength, and excellent tissue interface affinity, yet it is typically an electrical insulator in its pure state.

Rapid advancements in 3D bioprinting and biomanufacturing technologies, especially extrusion-based Direct Ink Writing technology, provide innovative solutions for the aforementioned challenges [20,21,22,23,24,25,26,27]. DIW technology enables the precise deposition of bio-inks (often containing biomolecules, cells, or biocompatible materials) and functional biomaterial inks [28,29,30,31,32], facilitating the digitalized, customized fabrication of complex biological structures or biomedical devices. Its unique capability of performing in situ printing on non-planar, soft biological substrates creates unprecedented opportunities for realizing the seamless integration of electronic devices with biological tissues, holding promise for advancing the field of personalized bioelectronics [33,34]. To address the inherent trade-off between conductivity and flexibility often found in single materials, careful design and optimization of the composite system is essential. This approach successfully combines the favorable conductivity of PEDOT:PSS with the excellent flexibility and high-strain performance imparted by the PVA matrix. This makes the composite particularly suitable for the DIW fabrication of flexible strain sensors capable of withstanding large deformations.

## 2. Materials and Methods

### 2.1. Materials

The composite conductive material utilized in this research consists primarily of two components: PEDOT:PSS dispersed particles (purchased from Zhuhai Kaiwo Optoelectronic Technology Co., Ltd., Zhuhai, China, which impart electrical conductivity; and Polyvinyl alcohol (PVA) powder ($\bar{Mw}$ = 146–186 kDa; purchased from Sigma-Aldrich, St. Louis, MO, USA), which contributes to the material’s flexibility and high-strain performance.

### 2.2. Preparation of the Conductive Composite Ink

Initially, an aqueous PVA solution was prepared to serve as the base matrix. Specifically, 5.0 g of PVA powder was immersed in 95.0 g of deionized (DI) water and allowed to stand overnight to ensure thorough swelling. Subsequently, this mixture was placed in a thermostatic oil bath and stirred at 800 rpm for 3 h at 80 °C until the PVA completely dissolved, forming a visually uniform and transparent 5 wt.% PVA solution.

Next, the precursor for the composite conductive material was prepared. Precisely 0.6 g of the above-prepared 5 wt.% PVA solution was weighed and placed into a clean beaker. Subsequently, 0.04 g of PEDOT:PSS dispersed particles and 0.36 g of DI water were added to this solution. The beaker was placed on a magnetic stirrer (MS-H-ProT, Beijing, China) and continuously stirred at 500 rpm for 3 h at room temperature to ensure the uniform dispersion of PEDOT:PSS within the PVA matrix, yielding the initial PEDOT:PSS/PVA mixed solution.

Finally, the homogeneous mixed solution was subjected to freeze-drying to obtain the solid PEDOT:PSS/PVA composite material. The resulting solid was subsequently placed in an oven (DHG-9030A, Shanghai, China) and thermally annealed at 100 °C. The annealed solid PEDOT:PSS/PVA composite material was then redispersed in 0.93 g of deionized water to formulate the final conductive composite ink, in which PEDOT:PSS accounted for 4% and PVA for 3% (by weight), with a viscosity suitable for 3D printing. To eliminate air bubbles from the conductive ink, it was loaded into syringe barrels. The ink-loaded barrels were placed in a centrifuge (TG16, Shanghai, China) and centrifuged at 3000 rpm for 5 min to effectively remove entrapped air bubbles. The degassed ink was sealed and stored under refrigeration at 9 °C until further use. All formulated inks were freshly prepared and utilized for 3D printing within 1 day of their preparation.

### 2.3. In Situ Printing of Strain Sensor Circuits on the Hand

To achieve precise and conformal printing of strain sensors on specific regions of the hand, the target hand region was first scanned using a high-precision 3D scanner (Mini, Shenzhen, China) to obtain its detailed three-dimensional (3D) digital model. This 3D digital model was subsequently imported into CAD design software (Autodesk Fusion 360) and processed to generate a standardized model file in STL format suitable for subsequent design. A planar two-dimensional (2D) sensor circuit geometric pattern, pre-designed in the CAD software, was then imported into the virtual 3D space containing the hand model. Next, projection techniques were utilized to conformally map this 2D planar pattern onto the target curved surface of the hand model. This process generated a 3D sensor printing path that precisely matched the unique topology of the individual hand.

A series of spatial coordinate points defining the path was extracted from this 3D conformal path and exported as a coordinate data file. This coordinate file was manually post-processed and adjusted to generate an executable G-code file compatible with the extrusion-based 3D printer utilized (Bio-Architect^®^ SR, Hangzhou, China). Finally, this G-code file was loaded into the 3D printer control system. Following the G-code instructions, the printer precisely maneuvered the printhead along the predetermined path to deposit the material, thereby accomplishing the direct in situ fabrication of the flexible strain sensor on the target skin surface of the hand.

### 2.4. Strain Sensor Performance Characterization

To evaluate the sensing performance of the flexible strain sensors, the two electrode leads of the sensor were first electrically connected to the measurement ports of a digital LCR meter (TH2830, Changzhou, China) using flexible wires. The LCR meter was configured for real-time resistance (Ω) monitoring mode, and data were continuously recorded at a frequency of 5 Hz.

During the test, the finger joint of the subject wearing the sensor was sequentially flexed from a fully extended position (defined as 0°) to three predetermined angles: 30°, 60°, and 90°. The finger was held steady at each bending angle for 5 s, then returned to the fully extended position and held for another 5 s. This sequence constituted one complete test cycle.

After the experiments, the complete recorded time-series resistance data were exported in CSV file format. This raw data was subsequently used for data processing, quantitative analysis of sensor response characteristics, and results visualization.

### 2.5. Fourier Transform Infrared Spectroscopy Test

Fourier Transform Infrared (FTIR) spectroscopy is performed using an FTIR spectrometer (Thermo Scientific, NicoletiS20, Waltham, MA, USA) to identify the primary chemical functional groups in samples. The spectral acquisition parameters are typically set as follows: a scanning range of 4000–400 cm⁻^1^, a resolution of 4 cm⁻^1^, and signal accumulation over 32 scans to enhance the signal-to-noise ratio (SNR). Prior to sample analysis, an air background spectrum is first collected. This background spectrum is subsequently automatically subtracted during the sample’s spectral acquisition to effectively eliminate interference from ambient carbon dioxide, water vapor, and the instrumental background. Finally, various chemical functional groups present in the sample are accurately identified through detailed analysis of the position, intensity, and shape of characteristic absorption peaks in the resulting spectrum.

### 2.6. Scanning Electron Microscopy Test

The microstructural morphology of the samples is observed using a Scanning Electron Microscope (SEM) (Hitachi, TM4000Plus, Tokyo, Japan). For this analysis, a small quantity of the sample is carefully mounted onto a sample stub using conductive double-sided carbon tape. After the stub is loaded into the sample chamber, representative images of selected areas are acquired at an accelerating voltage of 15 kV and a magnification of 300×.

### 2.7. Tensile Test

The tensile mechanical properties of the samples are evaluated using a Universal Testing Machine (Instron 3365, Norwood, MA, USA). First, samples are prepared into rectangular strips, and their dimensions, such as length, width, and thickness, are precisely measured. During testing, both ends of each specimen are firmly and centrally secured in the grips of the testing machine. A uniaxial tensile load is then applied at a constant extension rate of 20 mm/min until the specimen fractures. Throughout this process, the testing machine records in real-time the applied load and the corresponding elongation of the gauge length. From these data, a typical stress–strain curve is generated.

### 2.8. Rheological Research

Rheological characterization of the samples is performed using a rotational rheometer (AR-G2, TA Instruments, New Castle, DE, USA) equipped with a 20 mm diameter steel parallel plate geometry set at a 400 µm gap. The shear storage modulus (G′) and loss modulus (G″) are determined at a constant frequency of 10 rad s⁻^1^ over a shear strain range of 100% to 100,000%.

### 2.9. Conductivity Measurement

Samples are cut into regular rectangular strips, and their width (*W*) and thickness (*T*) are accurately measured using Vernier calipers. The resistance (*R*) of the samples along their length (*L*) is measured using a digital bridge (TH2830, Changzhou Tonghui, Changzhou, China). To ensure accurate measurements, both ends of each sample strip are coated with conductive silver paste to establish good and stable ohmic contact with the test ports of the digital bridge. Finally, the conductivity (*σ*) is calculated from the measured resistance value and the sample’s geometric dimensions using the following formula:

σ=L/(R×W×T)

### 2.10. Biocompatibility Assessment

The animal experimental protocols in this study were approved by the Ethics Committee of Shenzhen Guangming District People’s Hospital (Ethics Approval No. LL-KT-2025070). After 3 days of direct contact between the PEDOT:PSS/PVA material and rat brain tissue, the animals were euthanized by an intraperitoneal overdose of Zoletil 50 (3–5 times the anesthetic dose). Death was confirmed by observing the absence of heartbeat, respiration, and corneal reflex for at least 10 min post-injection. Following euthanasia, brains were extracted and fixed by immersion in 4% paraformaldehyde solution for 12 h. The fixed brain tissues then underwent conventional graded alcohol dehydration, xylene clearing, and paraffin embedding, and were subsequently sectioned into 5 µm-thick serial coronal sections. Some sections were then stained with Hematoxylin and Eosin, while the remaining sections were stained with Masson’s trichrome, both according to standard operating procedures. All stained sections were observed under a light microscope, and images were captured for subsequent analysis.

## 3. Results

**Figure 1:**
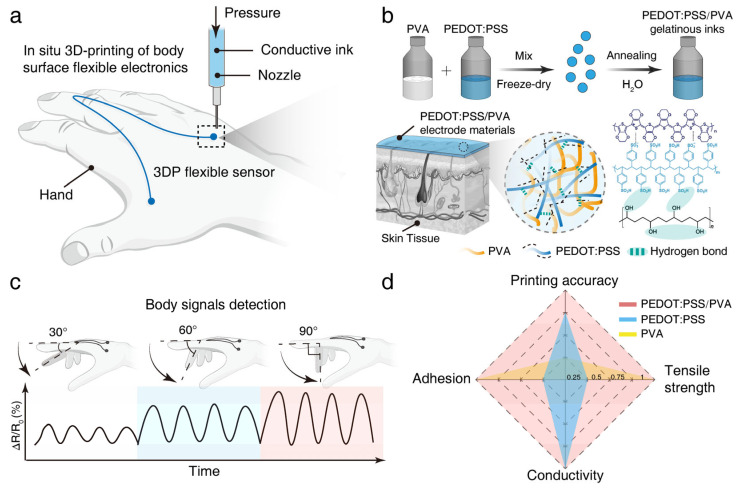
The 3D-printed strain sensor. (**a**) Schematic illustration of the process for in situ fabrication of a flexible sensor on the human hand skin surface (finger joint region) using DIW 3D printing (3DP) technology. (**b**) Preparation of the PEDOT:PSS/PVA composite material, illustration of conformal contact at the interface between the composite material and biological tissue, and a schematic of the material’s structure. The magnified region shows the interpenetrating network formed by the PEDOT:PSS conductive network and the PVA flexible matrix. (**c**) Application of the in situ 3D printed strain sensor for monitoring finger joint flexion–extension movements, showing the real-time response to different bending angles (30°, 60°, and 90°). Below are representative sensor signal waveforms corresponding to each angle. (**d**) Radar chart comparing key material properties (printing fidelity, skin adhesion, stretchability, and electrical conductivity) of the PEDOT:PSS/PVA composite ink with its primary components (PEDOT:PSS and PVA), where the numerical range of each axis on the radar chart represents the relative level of each property from lowest to highest.

As illustrated in Figure 1a, this study proposed and validated a strategy utilizing a PEDOT:PSS/PVA conductive composite ink and DIW 3D printing technology for the in situ fabrication of flexible strain sensors directly onto the human hand’s skin surface. The resulting device exhibits excellent conformal contact with the tissue (Figure 1b). Strain sensors fabricated in situ on the finger joint region were connected to a resistance measurement setup, and their signal responses were monitored as the finger executed bending motions of varying magnitudes (sequentially to 30°, 60°, and 90°), as shown in Figure 1c. Comparative analysis, potentially visualized using a radar chart, demonstrates that our material system effectively integrates desirable characteristics, including good printing fidelity, moderate electrical conductivity, and excellent mechanical stretchability. This demonstrates the significant advantages and feasibility of the proposed DIW printing strategy, combined with the optimized PEDOT:PSS/PVA composite ink, for the efficient and rapid fabrication of customized, high-performance on-body electronic devices. This research offers an innovative and promising new approach for developing next-generation wearable sensors that can be seamlessly integrated with the human body.

### 3.1. Design and Characterization of the 3D-Printable Conductive Composite Ink

**Figure 2:**
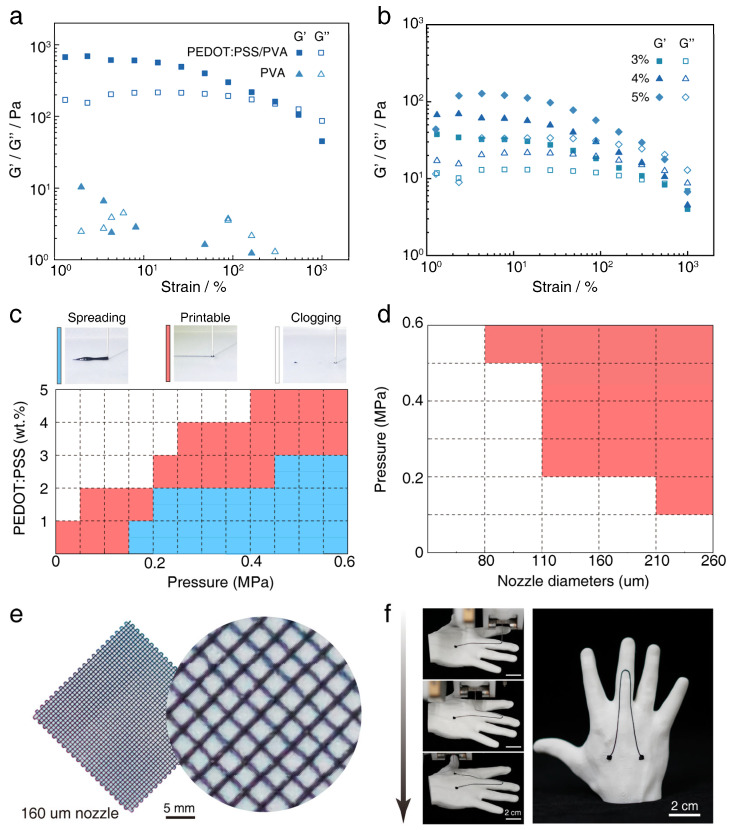
Design and characterization of the 3D-printable conductive composite ink. (**a**) Storage modulus (G′) and loss modulus (G″) as a function of strain for the PEDOT:PSS/PVA composite ink compared to the pure PVA solution. (**b**) Storage modulus (G′) and loss modulus (G″) as a function of strain for composite inks with different PEDOT:PSS concentrations (3 wt.%, 4 wt.%, and 5 wt.%). (**c**) Printability map constructed based on PEDOT:PSS concentration and printing pressure, where different colors or regions represent distinct printing outcomes (Spreading, Printable, Clogging). (**d**) Detailed printing process window map for the 4 wt.% PEDOT:PSS/PVA ink, constructed based on nozzle diameter and printing pressure. The specific region (red area) indicates the optimal parameter combinations for printing high-quality, continuous filaments. (**e**) Optical photograph of a precisely printed mesh structure using a 160 µm nozzle, demonstrating the ink’s high-resolution printing capability (Scale bar: 5 mm). (**f**) Photograph showing the sensor circuit pattern conformally printed onto a 3D hand model.

The successful implementation of DIW printing is highly dependent on the rheological properties of the ink. We investigated the ink’s properties using oscillatory rheology testing. Strain sweep results, shown in Figure 2a, reveal that compared to the pure PVA solution which behaves as a viscous fluid, the PEDOT:PSS/PVA composite ink exhibits a significantly increased storage modulus (G′) with G′ being much greater than the loss modulus (G″). This indicates the formation of a stable gel network structure conducive to shape retention after printing [35,36]. Concurrently, the composite ink displays a marked decrease in G′ in the high strain region, demonstrating typical shear-thinning behavior [37]. We further investigated the influence of PEDOT:PSS concentration on the rheological properties (Figure 2b). The results demonstrate that as the PEDOT:PSS concentration increased from 3 wt.% to 5 wt.%, both G′ and G″ of the ink systematically increased, reflecting an enhancement in the material’s viscosity and structural strength [38].

To determine the optimal ink formulation and printing process parameters, we constructed printability maps. By varying the PEDOT:PSS concentration and extrusion pressure, we systematically evaluated the printing outcomes. Figure 2c shows that excessively low concentrations or high pressures tended to cause ink spreading, whereas overly high concentrations or low pressures were prone to nozzle clogging. The ink containing 4 wt.% PEDOT:PSS exhibited the widest printable window within the tested parameter range (red region in Figure 2c) and was therefore selected as the optimal formulation. For this formulation, we further mapped the printability as a function of extrusion pressure and nozzle diameter (ranging from 80 µm to 260 µm). Figure 2d delineates the specific combinations of process parameters that enable the printing of continuous and uniform filaments (red region in Figure 2d).

To visually validate the high fidelity and conformal printing capability of the optimized 4 wt.% PEDOT:PSS/PVA ink and its associated printing process, we performed structural printing demonstrations. As shown in Figure 2e, using a 160 µm diameter nozzle, high-resolution mesh structures with well-defined, uniform lines were successfully printed, demonstrating the ink’s excellent printability and shape fidelity. Furthermore, we successfully printed the designed sensor circuit conformally onto the surface of a 3D hand model. Figure 2f clearly illustrates the ink’s ability to adapt well and adhere to complex curved surfaces, a key advantage for its application in direct on-body device printing. Collectively, these results confirm that this PEDOT:PSS/PVA composite ink is an excellent material suitable for high-precision, conformal DIW 3D printing.

### 3.2. Properties of the 3D-Printed Conductive Composite Material

**Figure 3:**
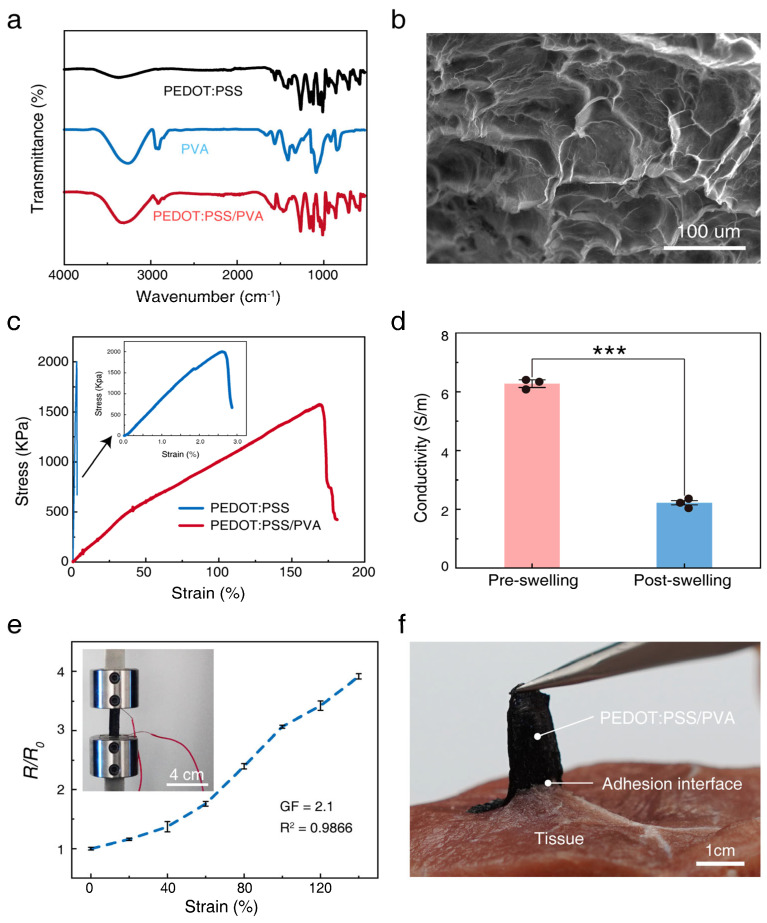
Properties of the 3D-printed conductive composite material. (**a**) Fourier Transform Infrared Spectroscopy spectra of pure PEDOT:PSS, pure PVA, and the PEDOT:PSS/PVA composite material. (**b**) Representative Scanning Electron Microscopy image of the PEDOT:PSS/PVA composite material. (**c**) Comparison of engineering stress–strain curves for pure PEDOT:PSS and the PEDOT:PSS/PVA composite material. The inset shows a magnified view of the PEDOT:PSS stress–strain curve. (**d**) Bar chart comparing the electrical conductivity of the PEDOT:PSS/PVA composite material before and after swelling. The asterisk (***) is used to indicate a statistically significant difference in electrical conductivity before and after swelling. Three independent replicate experiments were performed (*n* = 3). (**e**) Resistance response curve showing the relative resistance change (R/R_0_) of the PEDOT:PSS/PVA composite material as a function of applied strain. (**f**) Photograph of a simulated 90° peel test assessing the interfacial adhesion strength between the PEDOT:PSS/PVA material and biological tissue (Scale bar: 1 cm).

To investigate the composite effect between PEDOT:PSS and PVA, we first employed Fourier Transform Infrared Spectroscopy (FTIR) for chemical structure characterization. As shown in Figure 3a, the spectrum of pure PVA displays a typical broad absorption band at 3256 cm⁻^1^ (attributed to O-H stretching vibrations of hydroxyl groups) and exhibits its characteristic absorption peaks at positions including 2914 cm⁻^1^ (symmetric C-H stretching), 1411 cm⁻^1^ (CH-OH bending), 1327 cm⁻^1^ (C-H bending), and 1086 cm⁻^1^ (C-O stretching). The spectrum of pure PEDOT:PSS shows its characteristic absorptions at 1265 cm⁻^1^ (C-C stretching of the thiophene ring), 1161/1120 cm⁻^1^ (symmetric vibration of the sulfonate group SO_3_⁻ in PSS), 1059/1040 cm⁻^1^ (C-O-C stretching of the ethylenedioxy group), and in the region below 1000 cm⁻^1^ (e.g., 943, 858, 710 cm⁻^1^, attributed to C-S bond vibrations of the thiophene ring in PEDOT) [39]. Notably, the spectrum of the PEDOT:PSS/PVA composite material simultaneously contains the main characteristic absorption peaks originating from both PVA and PEDOT:PSS. This result clearly confirms the successful incorporation of both components within the composite material. Furthermore, we observed the material’s microstructure using Scanning Electron Microscopy (SEM). The SEM image in Figure 3b reveals a uniformly distributed porous microstructure within the composite material, indicating a homogeneous mixing of PEDOT:PSS and PVA at the microscale [40].

Possessing excellent mechanical tensile properties is critical for flexible strain sensors; therefore, we evaluated the mechanical performance of the PEDOT:PSS/PVA composite material through tensile testing. The stress–strain curve in Figure 3c indicates that the composite material exhibits remarkable flexibility and ductility, with an elongation at break reaching approximately 170%. This value far exceeds that of pure PEDOT:PSS material (shown in the inset of Figure 3c), demonstrating that the incorporation of PVA significantly enhances the material’s flexibility. This high stretchability provides a solid mechanical foundation for the sensor’s application in monitoring large-range human motions, such as joint flexion and extension [41,42,43,44].

In addition to mechanical properties, appropriate electrical conductivity is equally crucial for achieving effective sensing functionality. We measured the electrical conductivity of the composite material before swelling and after swelling. Figure 3d shows that the material possesses an electrical conductivity of approximately 6 S/m in its dry state. Although the conductivity decreases to approximately 2 S/m after swelling in moisture due to volumetric expansion from water absorption, this value remains sufficient for strain sensing applications based on resistance changes [45]. To further verify its sensing capability, we tested the relationship between the material’s relative resistance change (R/R_0_) and applied strain. The test results in Figure 3e clearly demonstrate a quasi-monotonic increasing trend of the R/R_0_ value with increasing strain, confirming that the composite material can effectively transduce mechanical deformation into measurable electrical signals.

Considering that the sensor is intended for direct application onto the skin surface, its interfacial adhesion to biological tissue is a core element for ensuring stable signal transmission and secure wear. We assessed the adhesion strength between the material and a tissue model using a simulated 90° peel test. Figure 3f shows that under peeling force, the PEDOT:PSS/PVA material undergoes significant deformation before detaching from the substrate, visually reflecting the formation of a strong interfacial bond between the two. This excellent adhesion performance is crucial for preventing sensor displacement or detachment during daily activities [46,47]. Although the interfacial bonding is strong, considering the hydrophilic nature of the PVA matrix, the sensor can be moistened with water before removal. This significantly reduces its adhesion to the skin, enabling a gentler peeling process to minimize user discomfort.

### 3.3. Application of the 3D-Printed Strain Sensor and Electrodes

**Figure 4:**
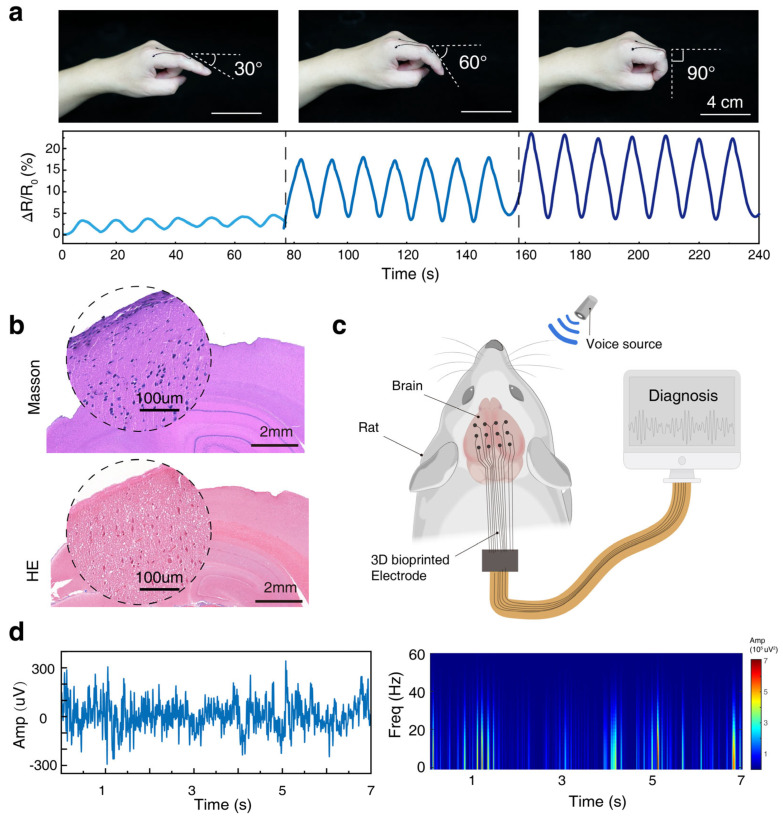
Application of the 3D-printed strain sensor and electrodes. (**a**) Performance of the on-body printed strain sensor monitoring finger joint movement: Top panel shows schematic positions during the finger bending test; bottom panel shows the real-time relative resistance change (ΔR/R_0_) response curves recorded by the sensor during cyclic bending to 30°, 60°, and 90°. (**b**) Biocompatibility assessment of the PEDOT:PSS/PVA composite material after contact with brain tissue: Representative microscopy images of histological sections stained with Masson’s trichrome and H&E (Scale bar: 2 mm). (**c**,**d**) Conceptual illustration of using the composite material to fabricate a flexible neural electrode array for monitoring neural electrical activity evoked by external stimuli (e.g., sound signals), including a schematic diagram, a representative time-amplitude curve, and a time-frequency heat map.

To validate the practical application performance of the flexible strain sensor printed directly onto the finger joint, we systematically evaluated its capability to monitor dynamic finger movements. As depicted in Figure 4a, we recorded the real-time relative resistance change (ΔR/R_0_) signals from the sensor as the finger performed cyclic flexion-extension movements towards different target angles (sequentially 30°, 60°, and 90°). The experimental data show that the in situ printed sensor not only responds stably and highly repeatably to each dynamic bending and straightening action of the finger, generating clear periodic resistance signal waveforms, but more importantly, its signal amplitude exhibits a clear positive correlation and stepwise increase with the joint bending angle. This result clearly demonstrates the sensor’s capability to quantitatively distinguish different degrees of bending.

To further objectively evaluate the performance of the strain sensor prepared in this work, we compared its key parameters with other flexible sensors reported in the literature (as shown in Table 1). This sensor exhibited a strain range of 170%, offering the possibility for large deformation monitoring. In terms of electrical conductivity, this sensor measured 2 S/m, which is close to the 2.04 S/m reported by Siti Aisyah Nurmaulia Entifar et al. This contributes to a stable signal response, whereas this parameter was not reported in detail for some other sensors. Regarding the GF value, this sensor’s GF was 2.1 (Figure 3e). For reference, the GF value of the hydrogel reported by Yulia Shara br Sembiring et al. was 0.58 [48], Siti Aisyah Nurmaulia Entifar et al. reported 1.034 [45], and Anky Fitrian Wibowo et al. reported 1.2 [49]. Higher GF values have also been reported in the literature, such as 31.2 by Gang Chen et al. [50], and a PEDOT:PSS sensor by Hao Liu et al. which reached a GF value of 2.61 under specific pre-stretching conditions [51]. The GF value of this sensor reflects its good strain sensitivity characteristics. Synthesizing these parameters, this strain sensor demonstrated coordinated performance in strain capability, conductive properties, and sensitivity, which supports its effective monitoring in diverse application scenarios and its broad potential in the future.

Considering the broad application potential of this composite material system in wearable and potentially future implantable bioelectronic devices, we conducted a preliminary histological evaluation of its biocompatibility. After placing the PEDOT:PSS/PVA material in direct contact with brain tissue for a defined period, we prepared tissue sections and performed Hematoxylin and Eosin (H&E) staining and Masson’s trichrome staining. The histological examination results in Figure 4b show that at the direct interface between the material and the brain tissue, the tissue maintained relatively intact cell morphology and organizational structure. No obvious signs of cell necrosis or large-scale inflammatory cell aggregation were observed in the H&E stained sections, nor did Masson’s staining reveal significant fibrous tissue proliferation (fibrosis). These preliminary histological findings suggest that the PEDOT:PSS/PVA composite material possesses good initial biocompatibility with biological tissue.

Combining the material’s demonstrated electrical conductivity, excellent flexibility, printability, and preliminarily established biocompatibility, this composite system shows significant potential for expanded applications in more complex bioelectronic interfaces, such as flexible neural electrode arrays. Future research could focus on utilizing DIW technology to achieve the in situ printing of high-resolution, customized neural electrodes onto the surface of target neural tissues, such as the cerebral cortex. This technology holds promise for the long-term, stable recording or modulation of neuronal activity, exemplified by the conceptual monitoring of sound-evoked brain electrical signals shown in Figure 4c,d. This study lays an important foundation in terms of materials and manufacturing methods for these challenging yet promising future application directions.

## 4. Conclusions

This study successfully developed and demonstrated an innovative strategy based on Direct Ink Writing biomanufacturing technology and a customized PEDOT:PSS/PVA composite bio-ink, achieving the direct, conformal, and in situ fabrication of functional flexible bioelectronic devices on the complex biological surface of human skin. Systematic engineering and optimization of the biomaterial ink’s rheological, mechanical, electrical, and bio-interfacial adhesion properties enabled this in situ printing method to effectively overcome the bio-integration challenges faced by conventional devices. This validated the feasibility and unique potential of this approach for manufacturing highly personalized wearable biomedical sensing devices that are tightly coupled with human tissues. The fabricated sensors were demonstrated to effectively monitor physiological biomechanical movements. While this study validated the sensor’s effectiveness in capturing such movements, a more detailed quantification of its dynamic response characteristics, such as precise response and recovery times, was not extensively performed due to limitations within the current experimental scope; this will be an important research direction in future work to further enhance sensor performance and expand its application potential in monitoring high-speed dynamic events. This research lays an important material and manufacturing foundation for the development of next-generation on-body bioelectronic interfaces.

## Figures and Tables

**Table 1:** Comparison of performance parameters for flexible strain sensors.

Materials	Strain Range (%)	Electrical Conductivity (S/m)	Gauge Factor	Ref.
ALG/PEDOT:PSS hydrogel	138	Not Reported	0.58	[48]
PEDOT:PSS/MWCNT	80	Not Reported	31.2	[50]
CMC-PVA-PEDOT:PSS	303.8	2.04	1.034	[45]
HEC/L-PEDOT:PSS	190	Not Reported	1.2	[49]
PEDOT:PSS	100	Not Reported	gauge factors of 2.61, 1.87, and 1.59 for the 30%, 50%, and 70% pre-strain groups	[51]
This work	170	2	2.1	

## Data Availability

All data generated or analyzed during this study are included in this published article.

## Abbreviations

The following abbreviations are used in this manuscript:

DIW	Direct Ink Writing
PVA	Polyvinyl alcohol
DI water	Deionized water
3D	three-dimensional
2D	two-dimensional
3DP	3D printing
G′	Storage modulus
G″	Loss modulus
FTIR	Fourier Transform Infrared Spectroscopy
SEM	Scanning Electron Microscopy
R/R_0_	relative resistance change
ΔR/R_0_	relative resistance change
H&E	Hematoxylin and Eosin
σ	Electrical conductivity
R	Resistance

## References

[1] Kim H., Kwon Y.T., Lim H.R., Kim J.H., Kim Y.S., Yeo W.H. (2021). Recent advances in wearable sensors and integrated functional devices for virtual and augmented reality applications. Adv. Funct. Mater..

[2] Li Y., Zheng L., Wang X. (2019). Flexible and wearable healthcare sensors for visual reality health-monitoring. Virtual Real. Intell. Hardw..

[3] Barfield W., Caudell T. (2001). Basic concepts in wearable computers and augmented reality. Fundamentals of Wearable Computers and Augmented Reality.

[4] Kong X.T., Luo H., Huang G.Q., Yang X. (2019). Industrial wearable system: The human-centric empowering technology in Industry 4.0. J. Intell. Manuf..

[5] Heikenfeld J., Jajack A., Rogers J., Gutruf P., Tian L., Pan T., Li R., Khine M., Kim J., Wang J. (2018). Wearable sensors: Modalities, challenges, and prospects. Lab Chip.

[6] Singh B., Kaunert C., Vig K., Gautam B.K. (2024). Wearable Sensors Assimilated With Internet of Things (IoT) for Advancing Medical Imaging and Digital Healthcare: Real-Time Scenario. Inclusivity and Accessibility in Digital Health.

[7] Chen Y., Zhang Y., Liang Z., Cao Y., Han Z., Feng X. (2020). Flexible inorganic bioelectronics. npj Flex. Electron..

[8] Fallegger F., Schiavone G., Lacour S.P. (2020). Conformable hybrid systems for implantable bioelectronic interfaces. Adv. Mater..

[9] Song E., Li J., Won S.M., Bai W., Rogers J.A. (2020). Materials for flexible bioelectronic systems as chronic neural interfaces. Nat. Mater..

[10] Park J., Kim H.W., Lim S., Yi H., Wu Z., Kwon I.G., Yeo W.H., Song E., Yu K.J. (2024). Conformal fixation strategies and bioadhesives for soft bioelectronics. Adv. Funct. Mater..

[11] Khan M.A., Saibene M., Das R., Brunner I., Puthusserypady S. (2021). Emergence of flexible technology in developing advanced systems for post-stroke rehabilitation: A comprehensive review. J. Neural Eng..

[12] Zhao J., Yang Y., Bo L., Qi J., Zhu Y. (2024). Research Progress on Applying Intelligent Sensors in Sports Science. Sensors.

[13] De Pasquale G., Ruggeri V. (2019). Sensing strategies in wearable bio-mechanical systems for medicine and sport: A review. J. Micromech. Microeng..

[14] Zhao C., Wang Y., Tang G., Ru J., Zhu Z., Li B., Guo C.F., Li L., Zhu D. (2022). Ionic flexible sensors: Mechanisms, materials, structures, and applications. Adv. Funct. Mater..

[15] Ochoa M., Rahimi R., Ziaie B. (2013). Flexible sensors for chronic wound management. IEEE Rev. Biomed. Eng..

[16] Chen W., Lin J., Ye Z., Wang X., Shen J., Wang B. (2024). Customized surface adhesive and wettability properties of conformal electronic devices. Mater. Horiz..

[17] Liang X., Chen G., Lin S., Zhang J., Wang L., Zhang P., Wang Z., Wang Z., Lan Y., Ge Q. (2021). Anisotropically Fatigue-Resistant Hydrogels. Adv. Mater..

[18] Liang X., Chen G., Lin S., Zhang J., Wang L., Zhang P., Lan Y., Liu J. (2022). Bioinspired 2D Isotropically Fatigue-Resistant Hydrogels. Adv. Mater..

[19] Liang X., Chen G., Lei I., Zhang P., Wang Z., Chen X., Lu M., Zhang J., Wang Z., Sun T. (2022). Impact-Resistant Hydrogels by Harnessing 2D Hierarchical Structures. Adv. Mater..

[20] Zhang Y.H.P., Sun J., Ma Y. (2017). Biomanufacturing: History and perspective. J. Ind. Microbiol. Biotechnol..

[21] Zhang B., Luo Y., Ma L., Gao L., Li Y., Xue Q., Yang H., Cui Z. (2018). 3D bioprinting: An emerging technology full of opportunities and challenges. Bio-Des. Manuf..

[22] Skardal A. (2018). Perspective: “Universal” bioink technology for advancing extrusion bioprinting-based biomanufacturing. Bioprinting.

[23] Arslan-Yildiz A., El Assal R., Chen P., Guven S., Inci F., Demirci U. (2016). Towards artificial tissue models: Past, present, and future of 3D bioprinting. Biofabrication.

[24] Santoni S., Gugliandolo S.G., Sponchioni M., Moscatelli D., Colosimo B. (2022). 3D bioprinting: Current status and trends—A guide to the literature and industrial practice. Bio-Des. Manuf..

[25] He Y., Mao T., Gu Y., Yang Y., Ding J. (2020). A simplified yet enhanced and versatile microfluidic platform for cyclic cell stretching on an elastic polymer. Biofabrication.

[26] Cui H., Nowicki M., Fisher J., Zhang L. (2017). 3D bioprinting for organ regeneration. Adv. Healthc. Mater..

[27] Ng W.L., Chan A., Ong Y.S., Chua C.K. (2020). Deep learning for fabrication and maturation of 3D bioprinted tissues and organs. Virtual Phys. Prototyp..

[28] He Y., Yu Y., Yang Y., Gu Y., Mao T., Shen Y., Liu Q., Liu R., Ding J. (2022). Design and aligner-assisted fast fabrication of a microfluidic platform for quasi-3D cell studies on an elastic polymer. Bioact. Mater..

[29] Gogoi D., Kumar M., Singh J. (2024). A comprehensive review on hydrogel-based bio-ink development for tissue engineering scaffolds using 3D printing. Ann. 3D Print. Med..

[30] Yao Z., Feng X., Wang Z., Zhan Y., Wu X., Xie W., Wang Z., Zhang G. (2024). Techniques and applications in 3D bioprinting with chitosan bio-inks for drug delivery: A review. Int. J. Biol. Macromol..

[31] Fatimi A., Okoro O.V., Podstawczyk D., Siminska-Stanny J., Shavandi A. (2022). Natural hydrogel-based bio-inks for 3D bioprinting in tissue engineering: A review. Gels.

[32] Saunders R.E., Derby B. (2014). Inkjet printing biomaterials for tissue engineering: Bioprinting. Int. Mater. Rev..

[33] Tang H., Li Y., Chen B., Chen X., Han Y., Guo M., Xia H., Song R., Zhang X., Zhou J. (2022). In situ forming epidermal bioelectronics for daily monitoring and comprehensive exercise. ACS Nano.

[34] Ershad F., Patel S., Yu C. (2023). Wearable bioelectronics fabricated in situ on skins. npj Flex. Electron..

[35] Bian M., Jiang S., Liu S., Zhang L., Miao S., Zhou F., Zheng B. (2024). Fish gelatin and gellan gum mixture as edible ink for 3D printing. J. Food Eng..

[36] Yu H., Chi S., Li D., Wang L., Wang Y. (2022). Effect of gums on the multi-scale characteristics and 3D printing performance of potato starch gel. Innov. Food Sci. Emerg. Technol..

[37] Amorim P.A., d’Ávila M.A., Anand R., Moldenaers P., Van Puyvelde P., Bloemen V. (2021). Insights on shear rheology of inks for extrusion-based 3D bioprinting. Bioprinting.

[38] Wang F., Xue Y., Chen X., Zhang P., Shan L., Duan Q., Xing J., Lan Y., Lu B., Liu J. (2024). 3D printed implantable hydrogel bioelectronics for electrophysiological monitoring and electrical modulation. Adv. Funct. Mater..

[39] Wang X., Feng G., Li M., Ge M. (2019). Effect of PEDOT: PSS content on structure and properties of PEDOT: PSS/poly (vinyl alcohol) composite fiber. Polym. Bull..

[40] Liu Q., Qiu J., Yang C., Zang L., Zhang G., Sakai E. (2021). High-performance PVA/PEDOT: PSS hydrogel electrode for all-gel-state flexible supercapacitors. Adv. Mater. Technol..

[41] Huang Y., Zhao Y., Wang Y., Guo X., Zhang Y., Liu P., Liu C., Zhang Y. (2018). Highly stretchable strain sensor based on polyurethane substrate using hydrogen bond-assisted laminated structure for monitoring of tiny human motions. Smart Mater. Struct..

[42] Zhou Z., He Z., Yin S., Xie X., Yuan W. (2021). Adhesive, stretchable and antibacterial hydrogel with external/self-power for flexible sensitive sensor used as human motion detection. Compos. Part B Eng..

[43] Zhao R., Zhao Z., Song S., Wang Y. (2023). Multifunctional conductive double-network hydrogel sensors for multiscale motion detection and temperature monitoring. ACS Appl. Mater. Interfaces.

[44] Ma Y., Zhang D., Wang Z., Zhang H., Xia H., Mao R., Cai H., Luan H. (2023). Self-adhesive, anti-freezing MXene-based hydrogel strain sensor for motion monitoring and handwriting recognition with deep learning. ACS Appl. Mater. Interfaces.

[45] Entifar S.A.N., Entifar N.A.E., Wibowo A.F., Kim J.H., Sembiring Y.S., Saputro J.M.W., Kim H.-G., Kim J.-O., Xie G., Oh J. (2025). Extremely-low electrical-hysteresis hydrogels for multifunctional wearable sensors and osmotic power generators. Chem. Eng. J..

[46] Zhang Y., Wang C., Wang X., Yin M., Wang K., Zhou D., Zheng H., Yu S., Li S., Chen K. (2024). Mechanical robust, adhesive, self-healable and biodegradable protein-based electronic skin sensors for smart elderly care. Chem. Eng. J..

[47] Gao Y., Yu L., Yeo J.C., Lim C.T. (2020). Flexible hybrid sensors for health monitoring: Materials and mechanisms to render wearability. Adv. Mater..

[48] Sembiring Y.S., Vo T.T.T., Entifar S.A.N., Wibowo A.F., Kim J.H., Entifar N.A.E., Kim J.H., Baek S.W., Lee S.I., Kim M.S. (2025). Recyclable, Conductive Alginate-Based Hydrogels with High Stretchability and Low Electrical Hysteresis for Wireless Wearable Sensors. J. Mater. Chem. A.

[49] Wibowo A.F., Nagappan S., Entifar S.A.N., Kim J.H., Sembiring Y.S., Han J., Oh J., Xie G., Lee J., Kim J. (2024). Recyclable, ultralow-hysteresis, multifunctional wearable sensors based on water-permeable, stretchable, and conductive cellulose/PEDOT: PSS hybrid films. J. Mater. Chem. A.

[50] Chen G., Li Y., He P., Wei Y., Song J., Peng B., Li Y. (2025). Construction of dual conductive networks based on material jetting for high-performance flexible strain sensors. Addit. Manuf..

[51] Liu H., Zhang S., Li Z., Lu T.J., Lin H., Zhu Y., Ahadian S., Emaminejad S., Dokmeci M.R., Xu F. (2021). Harnessing the wide-range strain sensitivity of bilayered PEDOT: PSS films for wearable health monitoring. Matter.

